# IgG4-Related Disease

**DOI:** 10.1155/2013/532612

**Published:** 2013-01-16

**Authors:** John H. Stone, John K. C. Chan, Vikram Deshpande, Kazuichi Okazaki, Hisanori Umehara, Yoh Zen

**Affiliations:** ^1^Rheumatology Unit, Division of Rheumatology, Allergy and Immunology, Massachusetts General Hospital, Yawkey 2, 55 Fruit Street, Boston, MA 02114, USA; ^2^Department of Pathology, Queen Elizabeth Hospital, Wylie Road, Kowloon, Hong Kong; ^3^Department of Pathology, Massachusetts General Hospital, Warren 2, 55 Fruit Street, Boston, MA 02114, USA; ^4^Department of Gastroenterology and Hepatology, Kansai Medical University, Japan; ^5^Division of Hematology and Immunology, Department of Internal Medicine, Kanazawa Medical University, 1-1 Daigaku, Uchinada-machi, Kahoku-gun, Ishikawa 920-0293, Japan; ^6^Histopathology Section, Institute of Liver Studies, King's College Hospital, Denmark Hill, London SE5 9RS, UK

Over the past decade and with increasing pace in the last few years, a “new” disease has emerged, gradually affecting a wide range of medical specialties and explaining a host of conditions previously regarded as separate entities. This newly recognized condition is IgG4-related disease (IgG4-RD), a potentially multiorgan disorder that is characterized by elevated serum IgG4 concentrations in the majority of cases. IgG4-RD was recognized in modern times in Japan through a series of seminal observations that occurred during the 1990s and the first few years of this century [1–5], but it is clear in reviewing the medical literature that IgG4-RD has been present and reported upon in various guises going back at least to the 1800s [6–11]. 

In addition to the frequent elevations of serum IgG4 concentrations, certain major pathologic hallmarks are generally present to one degree or another across all organ systems, providing the principal foundation for the belief that the disparate organ manifestations associated with this diagnosis are in fact part of the same systemic disease. These pathologic features include a lymphoplasmacytic infiltrate with a high percentage of plasma cells within the lesion staining for IgG4; a peculiar pattern of fibrosis known as “storiform” fibrosis; a tendency to affect veins in a manner that leads to obliterative phlebitis; and mild to moderate tissue eosinophilia [12]. 

IgG4-RD appears to sit at an intersection between different inflammatory pathways. Many but not all patients have substantial allergic or atopic histories, and early indications are that a “modified” Th2 response is critical to this condition [13]. Other patients also develop tumefactive lesions leading to misdiagnoses of cancer. Still others have clinical manifestations and serological findings that lead to erroneous classifications of their diagnoses as “connective tissue diseases.” The full links between the various inflammatory players in this symphony of inflammation remain to be fully elucidated. It is likely that a broader understanding of the ways in which B and T cells, fibroblasts, plasma cells, immune complexes, and other elements interact in IgG4-RD will provide important insights into the nature of its individual inflammatory constituents and the broader immune system. 

IgG4-RD is now recognized as a worldwide disease [14]. The international community convened in Boston in 2011 to compare notes, share experiences, and plan ways for moving ahead in understanding this condition. Building upon crucial earlier work in Japan, consensus papers pertaining to the nomenclature of this condition and to its pathological features have been published [12, 15]. Japanese investigators have also published diagnostic criteria for IgG4-RD [16].

In this special issue, we are pleased to present more than two dozen papers on IgG4-RD that address a number of facets of this condition: from its clinical manifestations to its radiologic features; from its pathology hallmarks to its serologic characteristics; and from its diagnostic challenges to early indications of treatment success. These papers capture the essence of IgG4-RD in 2012 and represent the current state-of-the-art against which future advances will be compared.

*John H. Stone* *John H. Stone* *John K. C. Chan* *John K. C. Chan* *Vikram Deshpande* *Vikram Deshpande* *Kazuichi Okazaki* *Kazuichi Okazaki* *Hisanori Umehara* *Hisanori Umehara* *Yoh Zen* *Yoh Zen*
